# Cold- versus warm-season-forced variability of the Kuroshio and North Pacific subtropical mode water

**DOI:** 10.1038/s41598-022-26879-4

**Published:** 2023-01-05

**Authors:** Yuma Kawakami, Hideyuki Nakano, L. Shogo Urakawa, Takahiro Toyoda, Kei Sakamoto, Goro Yamanaka, Shusaku Sugimoto

**Affiliations:** 1 Department of Atmosphere, Ocean, and Earth System Modeling Research, Meteorological Research Institute, Tsukuba, Japan; 2 Department of Geophysics, Graduate School of Science, Tohoku University, Sendai, Japan

**Keywords:** Ocean sciences, Climate sciences

## Abstract

The ocean responds to atmospheric variations. Changes in sea surface winds, surface air temperature, and surface air humidity cause upper ocean variability by modulating air-sea momentum and heat exchanges. Upper ocean variability in the mid-latitudes on inter-annual and longer timescales has previously been considered to be attributable to atmospheric variations in the cold season, because atmospheric forcing is stronger in the cold season than in the warm season. However, this idea has not been sufficiently confirmed yet. Although the ocean model is a useful tool to evaluate the impact of the atmospheric forcing in each season, there are no past studies having examined ocean model responses respectively to the cold- and warm-season atmospheric forcing. In this study, we performed numerical experiments with an eddy-resolving ocean general circulation model and investigated oceanic responses to cold- and warm-season atmospheric forcing, focusing on the Kuroshio and North Pacific subtropical mode water (STMW) in the western mid-latitude North Pacific. We found that temporal variations of net Kuroshio transport and STMW distribution/temperature are dominantly controlled by atmospheric forcing in the cold season. These results suggest that cold-season atmospheric variations are key to obtaining insights into large-scale upper ocean variability in the North Pacific subtropical gyre.

## Introduction

Temporal changes in atmospheric conditions such as sea surface winds, surface air temperature, and surface air humidity cause upper ocean variability by modulating momentum and heat exchanges between the atmosphere and ocean. In the North Pacific, atmospheric fields differ greatly between the cold and warm seasons. In the cold season, the development of the Aleutian Low in the northern North Pacific leads to strong sea surface winds and intense sea surface cooling^1^. In contrast, in the warm season, the North Pacific Subtropical High, which is centered in the eastern North Pacific, covers the North Pacific widely, forming relatively calm conditions.

**Figure 1:**
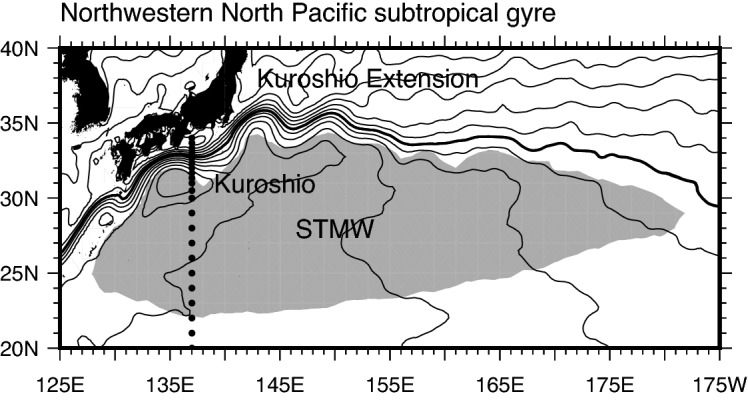
The Kuroshio and STMW in the northwestern North Pacific subtropical gyre. Contours represent mean sea surface height (cm) during 1993–2020 (10-cm intervals) (see text for details of the dataset). The 100-cm contour (thick line) is located near the Kuroshio axis south of Japan. The distribution region of STMW is schematically illustrated with gray shading based on Oka and Qiu^3^. Dots indicate the grid points of the optimally interpolated gridded dataset of the repeat hydrographic section along 137°E by the Japan Meteorological Agency (see text for details).

In the western North Pacific subtropical gyre, the Kuroshio flows poleward as the western boundary current, and North Pacific subtropical mode water (STMW)^2,3^ originated from the deep winter mixed layer (ML) south of the Kuroshio Extension (KE) is distributed widely (Fig. 1). The Kuroshio transports large amounts of seawater and heat northward, and the STMW volume in the upper ocean is huge: therefore, these two physical structures are key to understanding 3-dimensional temperature and salinity distributions in the western North Pacific subtropical gyre. Many studies have examined temporal variations of the Kuroshio transport^4–6^ and STMW distribution/temperature^7,8^. Further, the influence of cold-season atmospheric forcing on the Kuroshio transport has been well discussed^9,10^. In particular, it has been pointed out that wind stress curl (WSC) variations in the central North Pacific associated with both Aleutian Low activity^6,11^ and North Pacific Subtropical High variations^6^ have significant impacts on the Kuroshio transport through oceanic baroclinic responses. Many studies have also shown that sea surface cooling in the cold season is an important factor in STMW formation^12,13^. It was shown that the ML south of the KE (i.e., STMW formation region) becomes deep during winter in response to surface heat fluxes from observational data^14^, and that the anomalously deep ML is attributable to accumulation of enhanced surface cooling by wintertime storms based on reanalysis data^15^. Variations of wind stress fields in the cold season associated with Aleutian Low activity also affect STMW formation by modulating the KE path state^7,8^ and upper ocean stratification down to the main thermocline^16^ through oceanic Rossby waves. STMW temperature is determined by a combination of sea surface cooling^12^ and entrainment of low-level cold water into the ML in association with ML deepening^16^ in the cold season. The influence of warm-season atmospheric forcing on the Kuroshio and on STMW has also been examined. For example, Akitomo et al.^17^ implied influences of warm-season atmospheric forcing on Kuroshio transport by showing that the Kuroshio transport fluctuates in response to annual-mean WSC variations over the North Pacific. Further, in 2005, sea surface heating in the warm season resulted in anomalously strong surface ocean stratification in the northwestern North Pacific subtropical gyre and inhibited ML deepening (i.e., STMW formation) in the following winter^18^.

The strong sea surface winds and related intense surface cooling in the cold season have led many researchers to attribute Kuroshio and STMW variability to cold-season atmospheric forcing. However, whether atmospheric forcing in the cold season is the dominant cause of their variability is still unclear. It is possible that WSC variations associated with North Pacific Subtropical High variations in the warm season also strongly influence Kuroshio transport and that sea surface heating in the warm season substantially affects STMW formation and its temperature through modulation of preconditioning upper ocean conditions near the sea surface (e.g., temperature and stratification) before the cold season. An ocean model is one of useful tools to evaluate the impact of the atmospheric forcing in each season. However, there are no past studies having examined ocean model responses respectively to the cold- and warm-season atmospheric forcing. In this study, to obtain insights into the relationship between upper ocean variability and atmospheric forcing, we investigated which season is primary responsible for the variability of Kuroshio transport and STMW distribution/temperature by performing sensitivity experiments to the cold- and warm-season atmospheric forcing with the high-resolution (horizontal grid spacing ~ 10 km), eddy-resolving North Pacific model (NP model)^19,20^ developed by the Meteorological Research Institute (MRI).

## Results

To examine the influences of atmospheric forcings in the cold and warm seasons on Kuroshio transport and STMW distribution/temperature, we conducted three model experiments covering 1978–2013 in which we imposed different atmospheric conditions (see “Methods” and Table S1). In the CTRL run, the NP model is driven by raw 3-hourly atmospheric forcing data; the COLD (WARM) run is identical to the CTRL run except that it is driven by 3-hourly climatological forcing during the warm (cold) season. In this study, the cold and warm seasons were defined as October–March and April–September, respectively.

### Kuroshio

#### Reproducibility of the Kuroshio and the KE in the NP model

**Figure 2:**
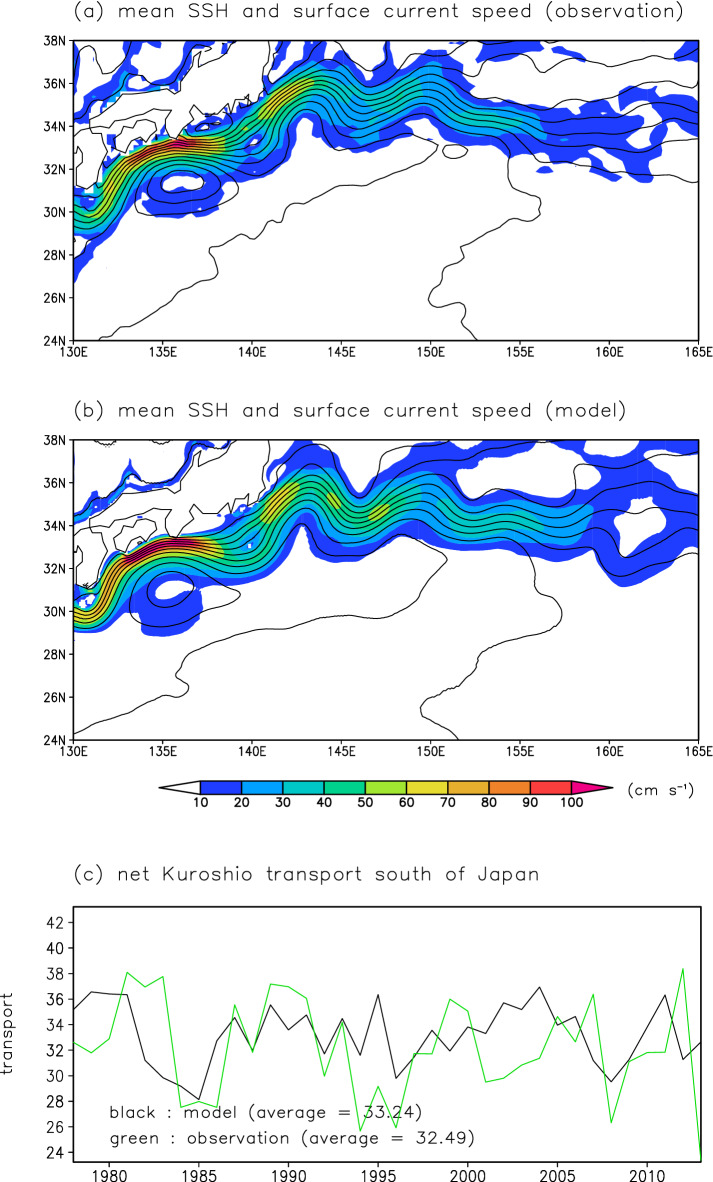
Reproducibility of the Kuroshio and the KE in the NP model. SSH (cm) (contour, 10-cm intervals) and surface current speed (cm s^–1^) (shading) from (**a**) satellite measurements and (**b**) the CTRL run averaged over 1993–2013. (**c**) Net Kuroshio transport (Sv; 1 Sv = 10^6^ m^3^ s^–1^) across 137°E from hydrographic observations by the Japan Meteorological Agency (green) and the CTRL run (black).

First, we checked the reproducibility of the Kuroshio and the KE in the NP model. In general, realistic simulation of the Kuroshio and the KE requires high-resolution settings^21,22^. The CTRL run results successfully reproduce the spatial distributions of mean sea surface height (SSH) and surface current velocity in the northwestern North Pacific subtropical gyre (Fig. 2a,b): the mean position and current speed of the Kuroshio and KE and of the Kuroshio recirculation south of Japan in the CTRL run are generally consistent with satellite measurements. The net Kuroshio transport across 137°E calculated in the CTRL run exhibits decadal-scale variation with positive peaks at around 1980, 1990, 2004, and 2011 and negative peaks at around 1985, 1996, and 2008 (Fig. 2c). This temporal behavior resembles that in observations^6^: positive and negative peaks in the simulation are consistent with the observational result, except in the early 2000s. The amplitudes of the observed and modeled net Kuroshio transport are similar, and their average values are almost the same.

#### Variability of the net Kuroshio transport

**Figure 3:**
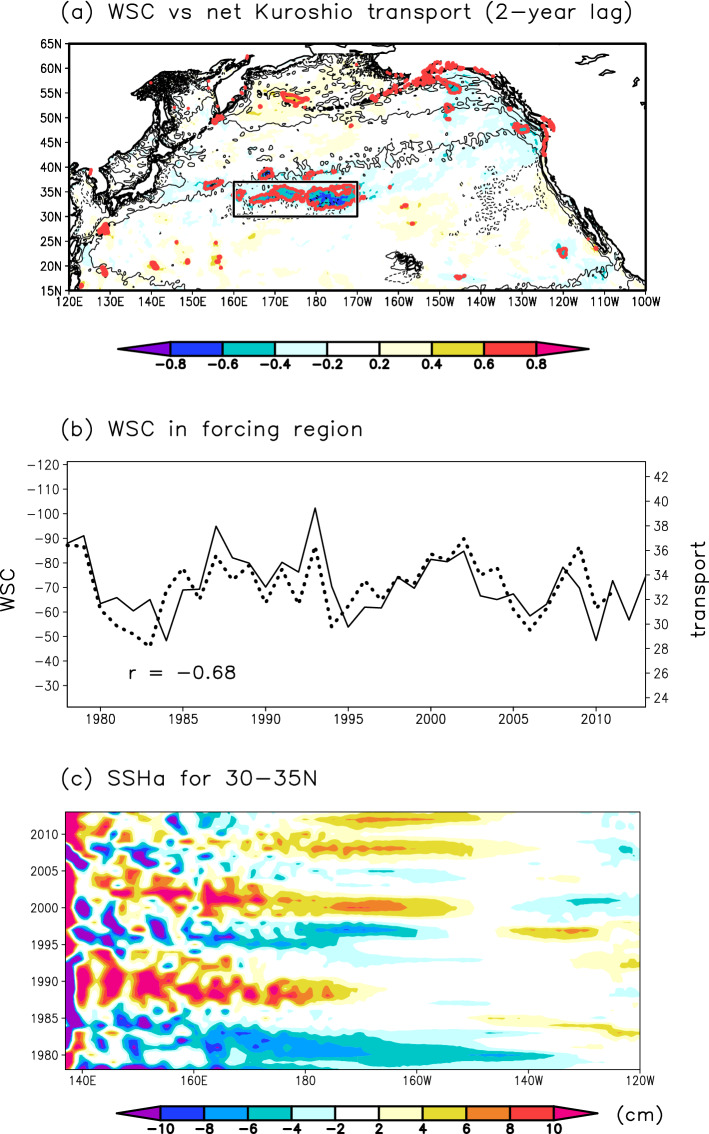
Relationship between the net Kuroshio transport and WSC fields in the North Pacific in the CTRL run. (**a**) Correlation coefficients between WSC and net Kuroshio transport with a 2-year lag (WSC leads net Kuroshio transport). Regions where the significance of the correlation coefficient exceeds the significance level of 0.1 are shown in red. Contours denote the WSC climatology (1978–2013) with 90 × 10^–9^ kg m^–2^ s^–2^ intervals (negative values are shown by dotted contours). The rectangle indicates the forcing region (30–37°N, 160°E–170°W). (**b**) WSC (10^–9^ kg m^–2^ s^–2^) (left axis) averaged over the forcing region. The dashed line indicates the net Kuroshio transport (Sv) (right axis) in the CTRL run shifted forward by 2 years. The correlation coefficient (*r*) between the two timeseries is shown in the lower left corner. (**c**) Longitude-time diagram of SSH anomalies (cm) averaged over 30–35°N from the CTRL run.

We next examined causes of the net Kuroshio transport variation by focusing on the influence of WSC variations over the North Pacific in the CTRL run. In this study, we used the annual-mean WSC to take both cold- and warm-season atmospheric forcings into account. Lag-correlation analysis results show that the net Kuroshio transport responds to WSC variations in the central North Pacific (30–37°N, 160°E–170°W; hereafter, the forcing region) with a 2-year lag (Fig. 3a,b). These forcing region and lag are consistent with the recent study^6^ based on hydrographic observations and atmospheric reanalysis data. It is notable that the significant correlations were not obtained with the other lags. Past studies have concluded that WSC variations in the central North Pacific cause net Kuroshio transport variations through westward propagation of oceanic Rossby waves^5,6,9,10^. In the NP model, the propagation speed of the baroclinic Rossby waves was estimated as about 2.5–3.5 cm s^–1^ at 30–35°N^20^; therefore, oceanic Rossby waves take 2–3 years to reach 137°E from around 155–172°E. Thus, the lag of the net Kuroshio transport to the WSC in the forcing region obtained from the above correlation analysis (i.e., 2 years) is consistent with oceanic Rossby wave propagation in the model. Evidence for oceanic Rossby waves is provided by a longitude-time diagram of SSH anomalies averaged over 30–35°N, which includes both the Kuroshio and the forcing region (Fig. 3c); the westward propagation from the formation region (i.e., the forcing region) of most signals in this diagram indicates oceanic Rossby waves, similar to observations (Fig. S1).

**Figure 4:**
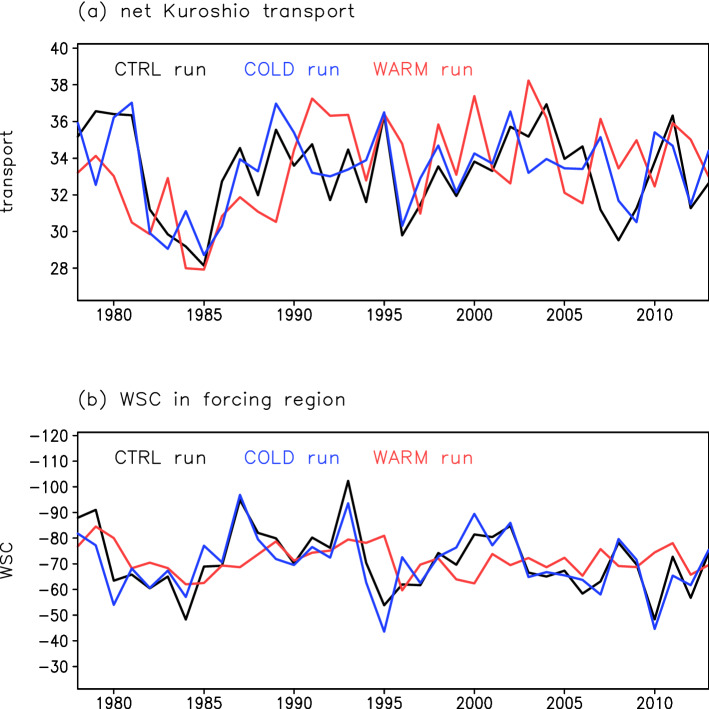
Temporal variations of net Kuroshio transport and WSC in the central North Pacific. (**a**) Black, blue, and red lines indicate net Kuroshio transport (Sv) across 137°E in the CTRL, COLD, and WARM runs, respectively. (**b**) Same as (**a**), but for WSC (10^–9^ kg m^–2^ s^–2^) in the forcing region (30–37°N, 160°E–170°W).

**Figure 5:**
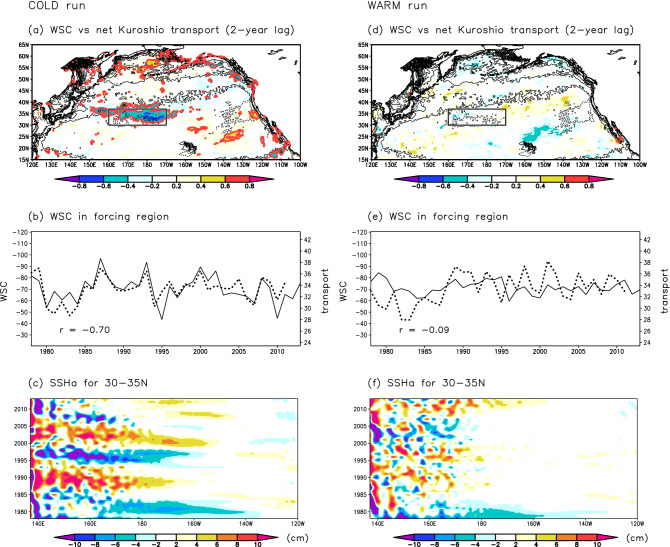
Relationship between the net Kuroshio transport and WSC fields in the North Pacific in the COLD and WARM runs. Same as Fig. 3, but for the (**a**–**c**) COLD and (**d**–**f**) WARM runs.

The net Kuroshio transport in the CTRL run is well reproduced in the COLD run (correlation coefficient (*r*) = 0.75, exceeding the significance level of 0.1) (Fig. 4a). The net Kuroshio transport in the COLD run fluctuated on a decadal timescale with almost the same amplitude as that in the CTRL run. In the COLD run, consistent with the CTRL run result, the transport is significantly correlated to WSC in the central North Pacific with a lag of 2 years (Fig. 5a,b). In the WARM run, however, the net Kuroshio transport fluctuated differently from that in the CTRL run (*r* = 0.35, non-significant), especially after 1990 (Fig. 4a). The net Kuroshio transport in the WARM run is not related to WSC variations over the North Pacific (Fig. 5d,e; see also Fig. S2) contrary to the CTRL run results. Westward propagation of oceanic Rossby waves from the central North Pacific is detectable in the COLD run, but not in the WARM run (Fig. 5c,f). In the WARM run, oceanic Rossby waves are not generated in the central North Pacific, and only small-scale SSH anomalies are detectable in the western region. These results indicate that oceanic Rossby waves that affect the net Kuroshio transport are generated by atmospheric forcing in the cold season. In fact, the amplitude of WSC variation in the central North Pacific is almost the same in the CTRL and COLD runs (about 15 × 10^–9^ kg m^–2^ s^–2^), and WSC values in the CTRL and COLD runs are significantly correlated (*r* = 0.88, exceeding the significance level of 0.1) (Fig. 4b). However, the WSC in the WARM run is less variable (amplitude of variation 5 × 10^–9^ kg m^–2^ s^–2^) and is not significantly correlated to that in the CTRL run (*r* = 0.32). Thus, our simulation results indicate that the WSC variation in the central North Pacific in the cold season dominantly drives the net Kuroshio transport variation. This result is robust evidence that supports the findings of past studies that focused on cold-season atmospheric forcing as the cause of net Kuroshio transport variation.

### STMW

#### Reproducibility of STMW in the NP model

**Figure 6:**
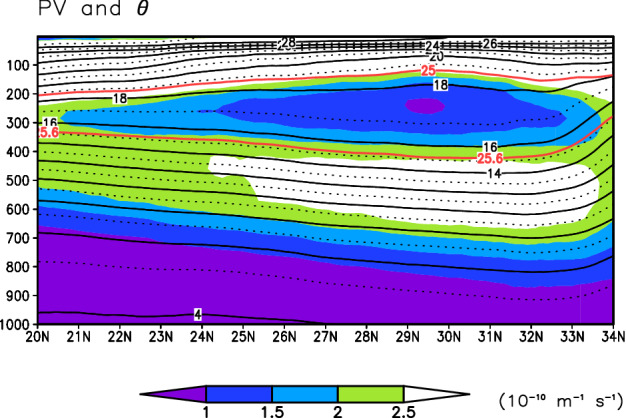
STMW properties in the NP model. Mean meridional-vertical cross section of PV (10^–10^ m^–1^ s^–1^) (shading) and *θ* (°C) (contours) in August for 1978–2013 averaged over 140–150°E in the CTRL run. Red lines indicate isopycnal surfaces of *σ*_*θ*_ = 25.0 and 25.6 kg m^–3^.

We investigate the sensitivity of STMW formation and temperature to cold- and warm-season atmospheric forcing. STMW in the northwestern North Pacific subtropical gyre is characterized by a vertically homogenous layer above the main thermocline and is detected as low-potential vorticity (PV) water. We first examined STMW properties in the CTRL run of the NP model. In August, STMW subducts into the subsurface. On the climatological meridional-vertical cross section of PV and potential temperature (*θ*) averaged over 140–150°E, low-PV water, representing STMW, with *θ* of 15–19 °C is centered at a depth of around 250 m between potential density (*σ*_*θ*_) of 25.0 and 25.6 kg m^–3^ (Fig. 6). This result is consistent with observational results^23,24^.

#### Variability of the STMW formation

**Figure 7:**
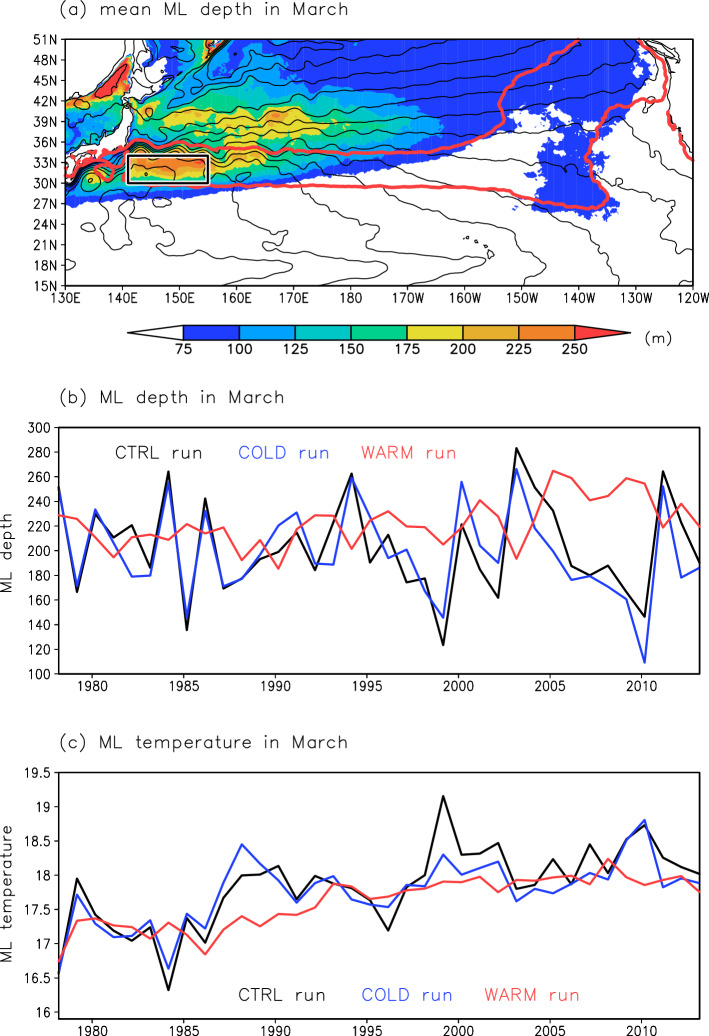
Temporal variations of the late-winter ML in the STMW formation region. (**a**) Distribution of ML depth (m) (shading) and SSH (cm) (contours, 10-cm intervals) in March averaged over 1978–2013 from the CTRL run. The rectangle indicates the STMW formation region (30–34°N, 141–155°E). Red lines indicate the mean positions of the isopycnal outcrops of *σ*_*θ*_ = 25.0 and 25.6 kg m^–3^ in March for 1978–2013. (**b**) Timeseries of ML depth (m) in March in the STMW formation region. Black, blue, and red lines represent the CTRL, COLD, and WARM runs, respectively. (**c**) Same as (**b**), but for ML temperature (°C) in March in the STMW formation region.

**Figure 8:**
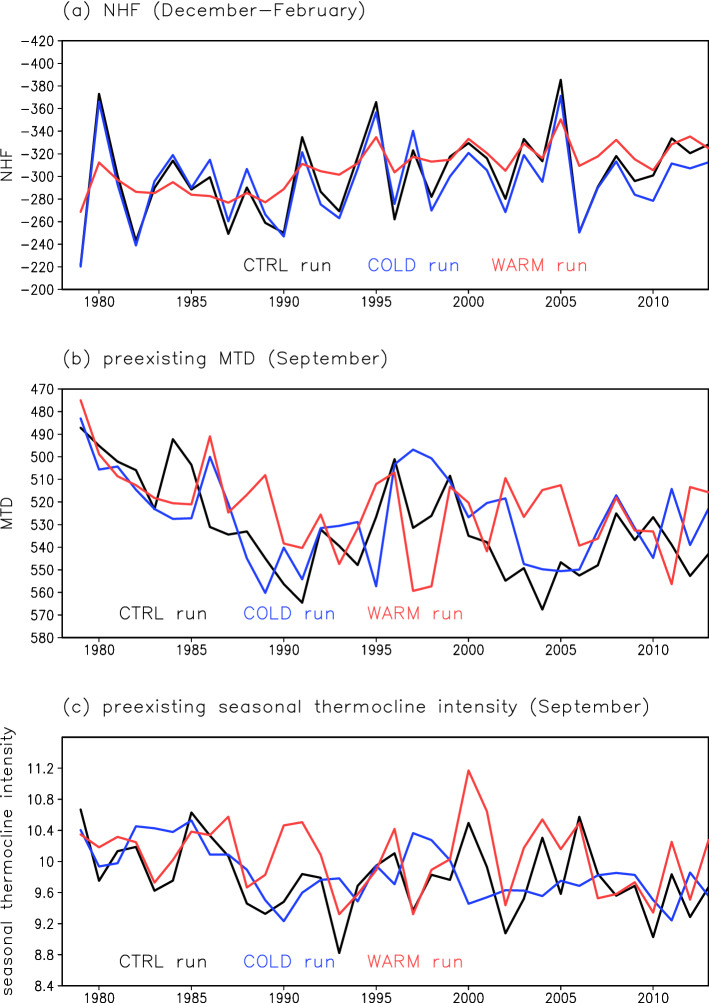
Temporal variations of the winter NHF, the preexisting MTD, and the preexisting seasonal thermocline intensity in the STMW formation region. (**a**) Mean NHF (negative values represent upward fluxes) (W m^–2^) for December–February, (**b**) MTD (m) in the preceding warm season (September), and (**c**) seasonal thermocline intensity (°C) in the preceding warm season (September) averaged in the STMW formation region. Black, blue, and red lines indicate the CTRL, COLD, and WARM runs, respectively. The MTD and seasonal thermocline intensity in the preceding warm season are plotted in the following year; e.g., the value in September 1999 is plotted in the year 2000.

We investigate temporal variations of STMW formation by focusing on the depth of the late-winter (March) ML in which STMW forms. In the CTRL run, the deepest climatological late-winter ML is found south of the KE between the outcrop regions of the isopycnal surfaces corresponding to STMW (*σ*_*θ*_ = 25.0–25.6 kg m^–3^) (Fig. 7a). Considering this ML depth distribution, we define the STMW formation region as 30–34°N, 141–155°E, consistent with observational results^3^. A timeseries of late-winter ML depth in the STMW formation region simulated by the CTRL run (Fig. 7b) shows decadal-scale variation with positive peaks at around 1984, 1994, and 2003, consistent with observations^7,16^. According to previous studies, the late-winter ML depth is controlled by two factors: the net surface heat flux (NHF) in winter and the preexisting upper ocean stratification^7,16,25^. As indicators of preexisting upper ocean stratification, we use the preexisting main thermocline depth (MTD) and the preexisting seasonal thermocline intensity. In the CTRL run, the winter (December–February) NHF in the STMW formation region shows inter-annual-scale variation (Fig. 8a), and the preexisting MTD shows decadal-scale variation (Fig. 8b). The seasonal thermocline intensity has inter-annual to decadal-scale variation (Fig. 8c). The results of a running correlation analysis confirmed that the winter NHF strongly influences the ML depth in the early and late parts of the analysis period (until 1993 and from 1997), and that the preexisting MTD has an important influence on the ML depth towards the end of the analysis period (from 1995) (Fig. S3). On the other hand, the preexisting seasonal thermocline intensity is not significantly correlated to the ML depth throughout the analysis period (Fig. S3). The preexisting upper ocean stratification crucial for the late-winter ML depth is the stratification down to the main thermocline rather than the stratification near the sea surface.

In the COLD run, the late-winter ML depth in the STMW formation region fluctuates on a decadal timescale (Fig. 7b); this temporal behavior is consistent with that in the CTRL run (*r* = 0.84, exceeding the significance level of 0.1). Fluctuations of the NHF and MTD simulated in the COLD run are also similar to those of the NHF and MTD, respectively, in the CTRL run (*r* = 0.96 for NHF and *r* = 0.56 for MTD, exceeding the significance level of 0.1) (Fig. 8a,b). In the WARM run, however, the ML depth variation is small (Fig. 7b), and its temporal features differ from those of the ML depth variation in the CTRL run (*r* = − 0.23, non-significant correlation). The temporal variations of the winter NHF in the WARM run are similar to those of the CTRL run (*r* = 0.77, exceeding the significance level of 0.1), but the amplitude of variation is much smaller (Fig. 8a). The fluctuations of the preexisting MTD in the WARM run differ from those in the CTRL run (*r* = 0.30, non-significant correlation) (Fig. 8b). The MTD variations in the North Pacific subtropical gyre have previously been attributed to oceanic Rossby waves caused by WSC variations in the central North Pacific^16^. Thus, the poor reproducibility of the MTD variation in the WARM run can be explained by a lack of oceanic Rossby wave signals (Fig. 5f) due to the small WSC variations in the central North Pacific (Fig. 4b). These results indicate that the late-winter ML depth in the STMW formation region (i.e., STMW formation) is determined mostly by cold-season atmospheric forcing, through surface cooling in the STMW formation region and surface wind stress in the central North Pacific.

We next investigate the summertime STMW distribution in the subsurface. The size of STMW area is calculated from the meridional-vertical cross section averaged over 140–150ºE in August, after STMW subduction (Fig. S4). The STMW area in the CTRL run shows a decadal size variation with positive peaks at around 1984, 1994, and 2004 that reflects the decadal variation of the late-winter ML depth in the STMW formation region (i.e., STMW formation) (Fig. 7b) (*r* = 0.39 for the correlation of the raw timeseries, non-significant correlation; *r* = 0.59 for the correlation of timeseries low-pass-filtered by a 1–3–4–3–1 filter, exceeding the significance level of 0.1). Decadal-scale variation in the size of the STMW area is also seen in the COLD run (*r* = 0.83 for the correlation with the STMW area variation in the CTRL run, exceeding the significance level of 0.1) (Fig. S4). In the WARM run, however, the variation in STMW area is much smaller than that in the CTRL run; this result reflects the small ML depth variation in the WARM run (Fig. 7b). Taken together, the COLD and WARM run results indicate that STMW formation and its distribution strongly reflect the cold-season atmospheric forcing.

#### Variability of the STMW temperature

We next focus on the late-winter ML temperature (Fig. 7c), from which STMW temperature originates^26^. Our simulation result confirms that the STMW core temperature (Fig. S4) reflects the late-winter ML temperature in the STMW formation region (*r* = 0.65 in the CTRL run, exceeding the significance level of 0.1). In the CTRL run, the late-winter ML temperature shows a decadal variation and a positive trend (0.038 °C year^–1^) in the STMW formation region (Fig. 7c). These features are similar to observational results^8,16,27^. An observational study^27^ has suggested that the surface warming trend around the Kuroshio region where STMW is formed is due to oceanic processes such as heat advection rather than to the surface heat flux. It has been also pointed out that the Atlantic Multidecadal Variability contributed to the surface warming in the STMW formation region from 1970 to 2010s through modulation of ocean heat advection in the western North Pacific^28,29^. Consistent with these results, in the CTRL run, the winter NHF shows no significant trend in the STMW formation region (Fig. 8a). It has also been demonstrated previously that decadal variation of the late-winter ML temperature is strongly influenced by entrainment of lower-level cold water into the ML in association with ML deepening in the cold season^16^. In the CTRL run, the detrended late-winter ML temperature is negatively correlated with the late-winter ML depth (*r* = − 0.60, exceeding the significance level of 0.1); this result indicates that an entrainment process plays an important role in the ML temperature variation. The late-winter ML temperature is not correlated with the winter NHF (*r* = 0.18, non-significant correlation), so the winter NHF variation cannot be the main cause of the ML temperature variation. However, the winter NHF indirectly affects the ML temperature through modulation of the ML depth. Similar to the CTRL run result, the ML temperature in the COLD run shows decadal variation and a warming trend (0.028 °C year^–1^) (Fig. 7c). The detrended ML temperatures in the CTRL and COLD runs are correlated (*r* = 0.84, exceeding the significance level of 0.1). However, the temporal features of the ML temperature in the CTRL run are not fully reproduced in the WARM run; the positive trend is reproduced (0.030 °C year^–1^), but not the decadal variation (Fig. 7c). These results indicate that the late-winter ML temperature variation in the STMW formation region is attributable to cold-season atmospheric forcing, which controls the late-winter ML depth variation. Furthermore, the STMW core temperature in subsurface layers is also strongly controlled by atmospheric forcing in the cold season (Fig. S4).

### Summary

Variability of the upper oceanic structure has been attributed to atmospheric forcing in the cold season, but whether atmospheric forcing in the cold season is really the dominant factor of upper ocean variability has not been clarified. To address this point objectively, we used an eddy-resolving ocean general circulation model that satisfactorily reproduces physical fields in the North Pacific and performed sensitivity experiments to cold- and warm-season atmospheric forcings.

Our simulations showed that net Kuroshio transport south of Japan is dominantly determined by WSC in the central North Pacific in the cold season. Through westward propagation of oceanic Rossby waves, net Kuroshio transport responds to WSC variations in the central North Pacific, which strongly reflect atmospheric fields in the cold season. Warm-season WSC variations in the central North Pacific are too small to generate oceanic Rossby waves, resulting in little influence on the net Kuroshio transport. The formation, distribution, and temperature of STMW also depend mostly on cold-season atmospheric forcing through surface cooling in the STMW formation region and surface wind stress in the central North Pacific. Warm-season atmospheric forcing with smaller variability than cold-season atmospheric forcing has less influence on the Kuroshio and on STMW.

The present study results suggest that cold-season atmospheric variations are key to obtaining insights into large-scale upper ocean variability in the North Pacific subtropical gyre, and they also support past researches that have considered the winter atmospheric forcing as the primary cause of upper ocean variability. These findings can guide future studies. Our approach is also applicable to other subtropical oceans worldwide.

## Methods

### Model experiments

We performed numerical simulations with the NP model^19,20^ developed by the MRI. This model is based on the MRI Community Ocean Model (MRI.COM)^30^, which is one of the standard ocean general circulation models used in the international intercomparison project (OMIP2)^31^. MRI.COM solves primitive equations under Boussinesq and hydrostatic approximations and adopts a vertically re-scaled height coordinate system in which sea level undulations are reflected throughout the water column^32^. The NP model domain is 15°S–63°N and 99°E–75°W (Fig. S5). The NP model has a horizontal resolution of 1/11° (longitude) × 1/10° (latitude) with 60 vertical levels; layer thickness increases with depth from 2 m at the top to 700 m for the lowest layer. The NP model is nested in a global ocean model with a horizontal resolution of 1° (longitude) × 1/2° (latitude) (GONDOLA_100)^33^ by a 2-way nesting method. The configuration of the NP model in this study was the same as that of previous studies^19,20^.

The atmospheric forcings used in this study (surface shortwave and longwave radiation fluxes, zonal and meridional wind speed at 10-m height, sea level pressure, precipitation, and air temperature and specific humidity at 10-m height) are from the 3-hourly JRA55-do dataset^34^. Surface wind stress, latent and sensible heat fluxes, and evaporation are calculated using bulk formulas^35,36^. The initial conditions were created by GONDOLA_100 driven by the JRA55-do dataset (1959–2013) for 5 cycles from the World Ocean Atlas 2013 climatology^37^; the restart of the 5th cycle at 00 UTC on 1 January 1978 was used for the initial conditions. The model experiments covered the 36-year period from 1978 to 2013 (CTRL run).

To examine the influences of cold- and warm-season atmospheric forcings on the Kuroshio transport and STMW distribution/temperature, we conducted two experiments in which different atmospheric conditions were imposed (summarized in Table S1). Cold and warm seasons were defined as October–March and April–September, respectively. The first experiment (COLD run) was conducted with raw 3-hourly JRA55-do forcing during the cold season and with 3-hourly climatological forcing (30-year average for 1981–2010) during the warm season. The second experiment (WARM run) was conducted similarly to the COLD run, but with raw JRA55-do forcing in the warm season and climatological forcing in the cold season. Here, using the climatological wind speed at 10-m height results in unrealistically small climatological surface wind stress because of the nonlinearity of the bulk formula. To avoid this problem, we constructed a dataset for wind stress forcing in the COLD and WARM runs using the 3-hourly output of the CTRL run, and we imposed these data directly.

### Observational data

To validate the model results, we used the net Kuroshio transport timeseries calculated from optimally interpolated (OI) grided temperature and salinity data of the repeat hydrographic section along 137°E maintained by the Japan Meteorological Agency^6^. The 137°E OI dataset is constructed from temperature and salinity profiles obtained with a reversing thermometer, Nansen bottles, a conductivity-temperature-depth (CTD) profiler with Niskin bottles, expendable bathythermographs, expendable CTD (XCTD), and a digital bathythermograph. The horizontal interval of grid points is 1/3° for 31–34°N, 1/2° for 30–31°N, and 1° for 3–30°N (see Fig. 1), and the vertical interval is 1 dbar. Winter (mainly January) and summer (mainly July–August) cruise data are available since 1967 and 1972, respectively. See reference paper^6^ for details of this dataset and calculation of the net Kuroshio transport.

We also used satellite-derived daily SSH and surface current velocity data from the Copernicus Marine Environment Monitoring Service. The horizontal resolution of these data is 1/4° (longitude) × 1/4° (latitude). These SSH and surface current velocity data are available from 1993.

### Calculation of the net Kuroshio transport

We calculated the net Kuroshio transport across 137°E; this longitudinal position was selected because long-term Kuroshio transport observations are available there, so simulation results can be compared with observational data. South of Japan, the Kuroshio is accompanied by two westward flows (see Fig. 1); one is the cold-core eddy transport north of the Kuroshio, and the other is the Kuroshio recirculation, that is, the Kuroshio counter current (KCC), south of the Kuroshio. In this study, we calculated annual-mean transports by the cold-core eddy, the Kuroshio, and the KCC following a method of the past work^5^. The northern and southern boundaries of the cold-core eddy, the Kuroshio, and the KCC were determined from the SSH distribution (Fig. S6), and their transports were calculated by vertical and meridional integration of the annual-mean zonal current velocity from the sea surface to 1000-dbar depth within each region. We defined the net Kuroshio transport as the sum of cold-core eddy transport, Kuroshio transport, and KCC transport.

### Definition of STMW

STMW is characterized by a vertically homogenous layer above the main thermocline and is detected as low PV water. Here, PV is calculated as follows:

1 $$\mathrm{PV}=-\frac{f}{ \rho }\frac{ \partial {\sigma }_{\theta } }{\partial z},$$

where *f* is the Coriolis parameter, *ρ* is the density of seawater, *σ*_*θ*_ is the potential density, and *z* is the vertical coordinate. The relative vorticity is ignored because the STMW distribution region is far from strong currents, near which the relative vorticity is comparable to the planetary vorticity. Using the meridional-vertical cross section averaged over 140–150°E in August, after subduction of STMW into the subsurface, we calculated the area and the core temperature of STMW. We defined the size of STMW area as the area of the region north of 20°N with PV < 1.5 × 10^–10^ m^–1^ s^–1^ and *θ* = 15–19 °C. We defined the STMW core temperature as *θ* at the PV minimum.

### Definition of the ML depth and the ML temperature

The ML depth was defined as the depth at which *σ*_*θ*_ increases by 0.03 kg m^–3^ from its value at the sea surface. The ML temperature was defined as *θ* at 10-m depth.

### Definition of the MTD and the seasonal thermocline intensity

The MTD was defined as the depth of the 12 °C isotherm, which is located near the center of the main thermocline in the North Pacific subtropical gyre (see Fig. 6). We checked that the 12 °C isotherm is located near the center of the main thermocline throughout the analysis period. The seasonal thermocline is formed between the sea surface and ~ 200-m depth with the center at around 50-m depth or shallower (see Fig. 6). The seasonal thermocline intensity was defined as the vertical *θ* difference between 1 and 200 m depths. We used the MTD and seasonal thermocline intensity in September as the preexisting MTD and the preexisting seasonal thermocline intensity.

### Significance test of correlation coefficients

In this study, we used the significance level of 0.1 for all correlation coefficients, based on Student’s two-sided t-test. The degrees of freedom were estimated by dividing the data length by the smallest lag for which an autocorrelation coefficient for either timeseries of interest becomes less than 0.1.

## Supplementary Information

Supplementary Information.

## Data Availability

All data are available from the corresponding author upon reasonable request. The OI dataset for the 137ºE section can be downloaded from the Japan Meteorological Agency website (http://www.data.jma.go.jp/gmd/kaiyou/db/mar_env/results/OI/137E_OI_e.html). The SSH product can be obtained from the Copernicus Marine Environment Monitoring Service website (https://marine.copernicus.eu).
