# Supplementary material for: Cold- versus warm-season-forced variability of the Kuroshio and North Pacific subtropical mode water
Source: Sci Rep. 2023 Jan 5;13:256. doi: 10.1038/s41598-022-26879-4 (PMC9816105; doi:10.1038/s41598-022-26879-4)
Supplement: Supplementary file 1 — Supplementary Information. [file 41598_2022_26879_MOESM1_ESM.docx]

Supplementary information for

**Cold- versus warm-season-forced variability of the Kuroshio**

**and North Pacific subtropical mode water**

Yuma Kawakami^1, *^, Hideyuki Nakano^1^, L. Shogo Urakawa^1^, Takahiro Toyoda^1^, Kei Sakamoto^1^, Goro Yamanaka^1^, and Shusaku Sugimoto^2^

^1^Department of Atmosphere, Ocean, and Earth System Modeling Research, Meteorological Research Institute, Tsukuba, Japan

^2^Department of Geophysics, Graduate School of Science, Tohoku University, Sendai, Japan

Contents of this file:

Table S1 and Figures S1 to S6

---

^*^Corresponding author: Yuma Kawakami (y-kawakami@mri-jma.go.jp)

Department of Atmosphere, Ocean, and Earth System Modeling Research, Meteorological Research Institute, 1-1 Nagamine, Tsukuba, Ibaraki 305-0052, Japan

**Table S1**

**Atmospheric forcings used in the model experiments**. The 30-year average during 1981–2010 was used as the climatology.

| Experiment | Forcing |
| --- | --- |
| CTRL run | 3-hourly raw data |
| COLD run | 3-hourly raw data (OCT–MAR) and 3-hourly climatology (APR–SEP) |
| WARM run | 3-hourly raw data (APR–SEP) and 3-hourly climatology (OCT–MAR) |

**
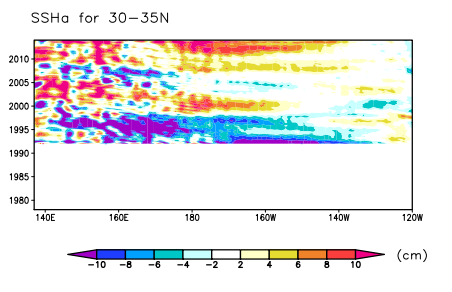
**

**Figure S1**

**Westward propagation of oceanic Rossby waves from satellite measurements.** Longitude-time diagram of SSH anomalies (cm) averaged over 30–35ºN from satellite observations.


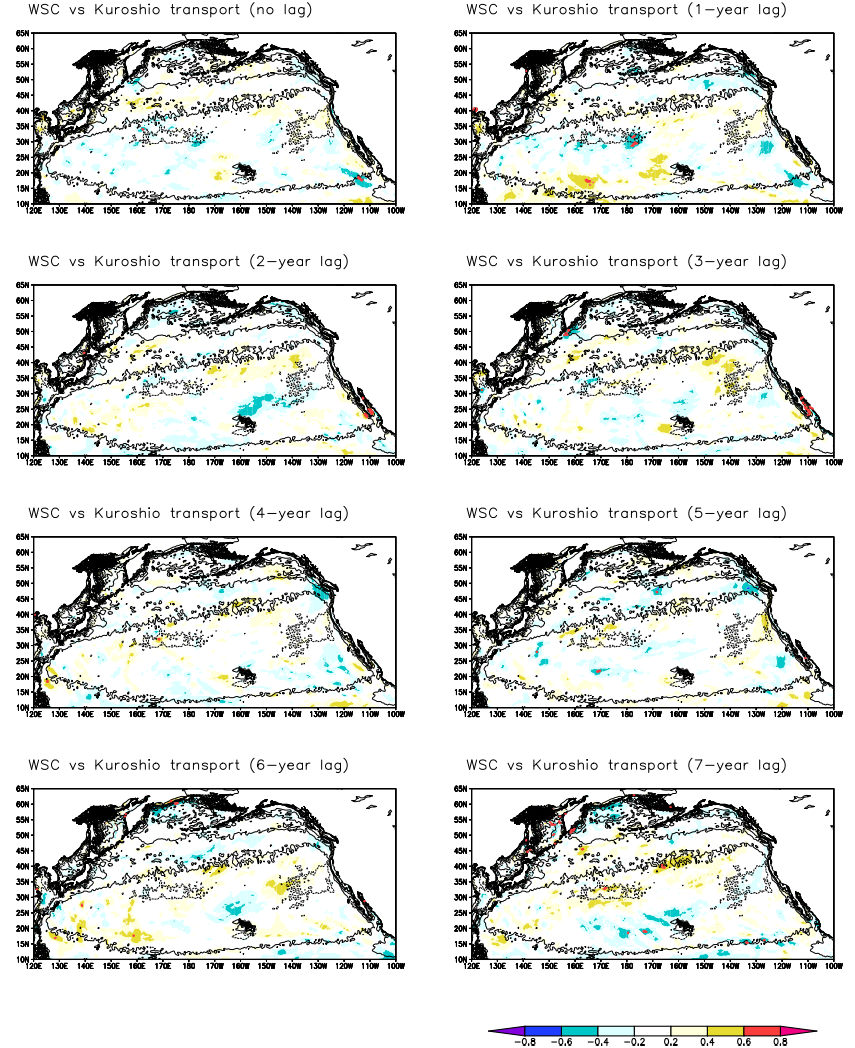


**Figure S2**

**Relationship between the net Kuroshio transport and WSC fields in the North Pacific with lags in the WARM run.** Correlation coefficients between WSC and the net Kuroshio transport with 0–7-year lags in the WARM run (WSC leads net Kuroshio transport). Regions where the significance of the correlation coefficient exceeds the significance level of 0.1 are shown in red. Contours denote the WSC climatology (1978–2013) with 90 × 10^–9^ kg m^–2^ s^–2^ intervals (negative values are shown by dotted contours).


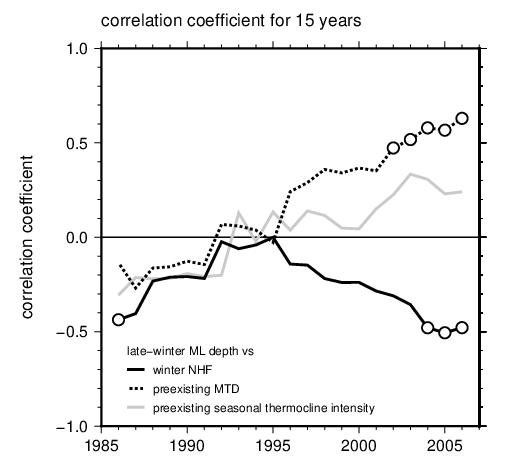


**Figure S3**

**Relationships of the late-winter ML depth in the STMW formation region to the winter NHF, the preexisting MTD, and the preexisting seasonal thermocline intensity.** The timeseries were obtained by a running correlation analysis (window size 15 years) of the late-winter ML depth vs the winter NHF (negative values indicate upward fluxes) (solid line), vs the MTD in the preceding warm season (September) (dotted line), or vs the seasonal thermocline intensity in the preceding warm season (September) (gray line) in the STMW formation region in the CTRL run. The correlation coefficient shown for a given year is calculated for the entire 15-year window; for instance, the correlation coefficient plotted at 1995 is that for 1988–2002. Open circles represent significant values exceeding the significance level of 0.1. All years in the analysis period except 1994 are included in at least one 15-year window in which the winter NHF and/or the preexisting MTD correlates significantly with the late-winter ML depth.


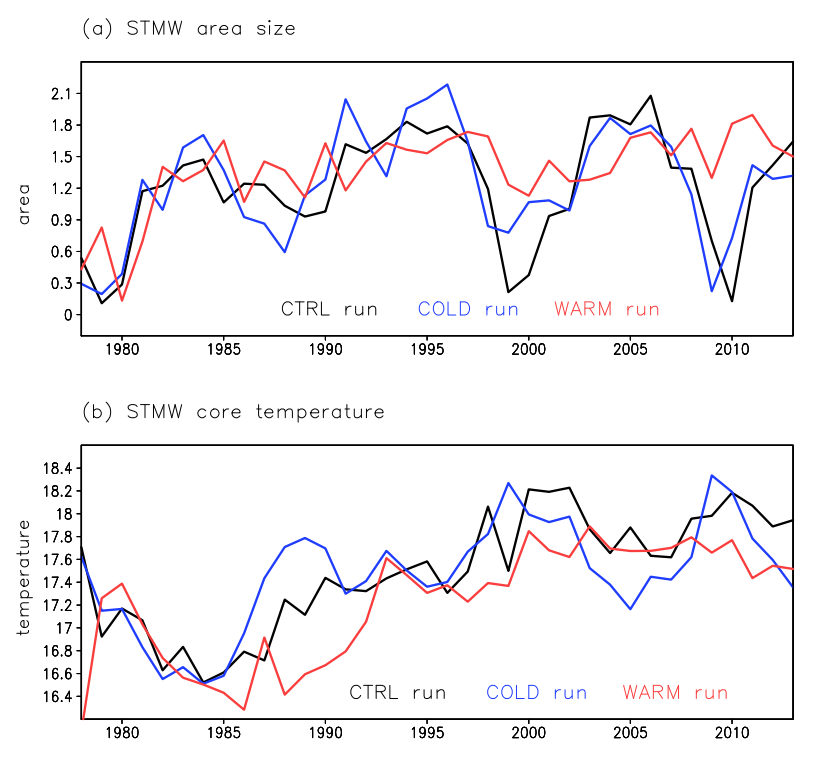


**Figure S4**

**Temporal variations of STMW area size and STMW core temperature.** (a) STMW area (10^8^ m^2^) and (b) STMW core temperature (℃) from a meridional-vertical cross section in August averaged over 140–150ºE. Black, blue, and red lines indicate the CTRL, COLD, and WARM runs, respectively.

**
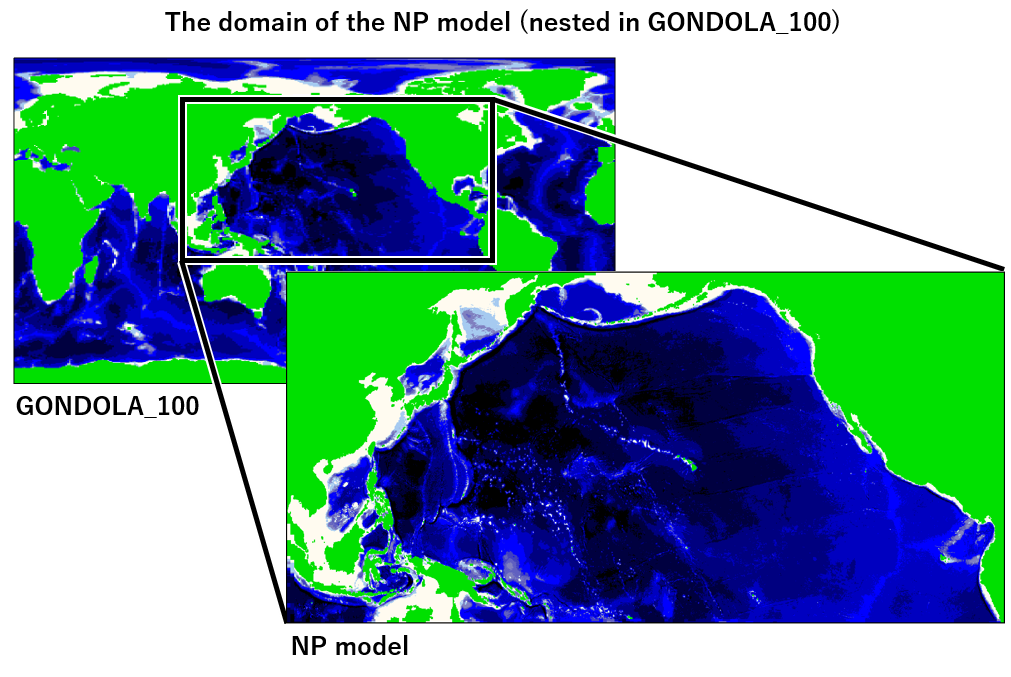
**

**Figure S5**

**The domain of the NP model.** The lower-right panel shows the NP model domain (15ºS–63ºN, 99ºE–75ºW). The NP model is nested within the global model GONDOLA_100 (upper-left panel; Urakawa et al.^33^) using 2-way nesting (see Methods for details).


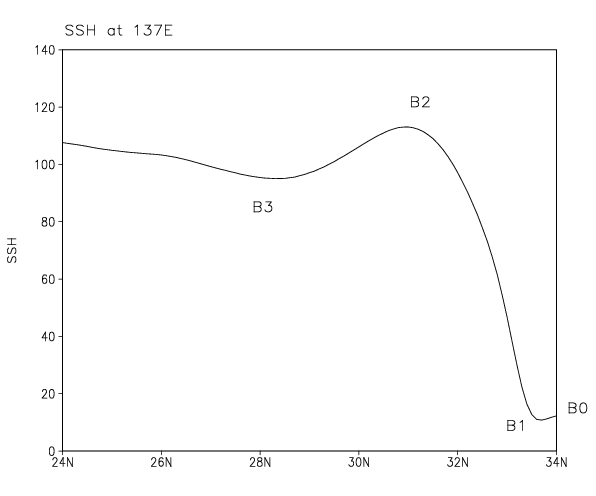


**Figure S6**

**Meridional distribution of SSH along 137ºE.** Schematic illustration of the SSH (cm) distribution. The westward flows of the cold-core eddy, the Kuroshio, and the KCC are defined as the flows between B0 and B1, between B1 and B2, and between B2 and B3, respectively.
